# Effects of Seasonal Variation on Spatial and Temporal Distributions of Ozone in Northeast China

**DOI:** 10.3390/ijerph192315862

**Published:** 2022-11-29

**Authors:** Jin Chen, Li Sun, Hongjie Jia, Chunlei Li, Xin Ai, Shuying Zang

**Affiliations:** 1 Heilongjiang Province Key Laboratory of Geographical Environment Monitoring and Spatial Information Service in Cold Regions, Harbin Normal University, Harbin 150025, China; 2 Heilongjiang Province Cold Region Ecological Safety Collaborative and Innovation Center, Harbin 150025, China

**Keywords:** O_3_ concentration, influencing factors, aggregation characteristics, geographical detector, Northeast China

## Abstract

The levels of tropospheric ozone (O_3_) are closely related to regional meteorological conditions, precursor emissions, and geographical environments, which have a significant negative impact on human health. The concentrations of O_3_ were relatively low, while the spatial distribution was strongly heterogeneous in Northeast China; however, little is known about how the influencing factors affect the distribution of O_3_ in Northeast China. Here, the O_3_ concentration, meteorological observation data, precursors (NO_2_), and vegetation coverage data from 41 monitoring cities in Northeast China from 2017 to 2020 were collected and analyzed. The spatial–temporal distributions and evolution characteristics of O_3_ concentrations were investigated using statistical analysis, kriging interpolation, spatial autocorrelation analysis, cold–hot spot analysis, and geographic detectors, and the effects of meteorological factors, NO_2_, and green land area on O_3_ concentrations were evaluated seasonally and spatially. The results showed that O_3_ pollution in Northeast China was generally at a relatively low level and showed a decreasing trend during 2017–2020, with the highest concentrations in the spring and the lowest concentrations in the autumn and winter. May–July had relatively high O_3_ concentrations, and the over-standard rates were also the highest (>10%). The spatial distribution showed that the O_3_ concentration was relatively high in the south and low in the northeast across the study area. A globally significant positive correlation was derived from the spatial autocorrelation analysis. The cold–hot spot analysis showed that O_3_ concentrations exhibited spatial agglomerations of hot spots in the south and cold spots in the north. In Northeast China, the south had hot spots with high O_3_ pollution, the north had cold spots with excellent O_3_ levels, and the central region did not exhibit strong spatial agglomerations. A weak significant negative correlation between O_3_ and NO_2_ indicated that the emissions of NOx derived from human activities have weak effects on the O_3_ concentrations, and wind speed and sunshine duration had little effect on spatial differentiation of the O_3_ concentrations. Spatial variability in O_3_ concentrations in the spring and autumn was mainly driven by temperature, but in the summer, the influence of temperature was weakened by the relative humidity and precipitation; no factor had strong explanatory power in the winter. The temperature was the only controlling factor in hot spots with high O_3_ concentrations. In cold spots with low O_3_ concentrations, the relative humidity and green land area jointly affected the spatial distributions of O_3_.

## 1. Introduction

Since the implementation of China’s Action Plan on Air Pollution Prevention and Control (2013), the concentrations of anthropogenic atmospheric pollutants such as SO_2_, NO_2_, CO, PM_2.5_, and PM_10_ have decreased significantly, illustrating the effectiveness of control measures on particulate pollution [1]. However, ozone (O_3_) pollution is still increasing [2]. According to the Bulletin of the Ecological Environment, the annual proportions of days with O_3_ levels exceeding the standard were 5.3%, 7.6%, 4.9%, and 4.4% from 2018 to 2021, respectively, making it the most important air pollutant after PM_2.5_. High concentrations of O_3_ seriously threaten the human cardiovascular and respiratory systems, damage the internal structures and ecological functions of plants, and even adversely affect global climate change [3,4,5]. O_3_ concentrations near the surface are dependent on precursor emissions [6], meteorological factors [7], chemical reactions [8], geographical environment [9], and regional transport [10] and generally show high spatial heterogeneity. Previous studies on the distributions of O_3_ have mainly focused on areas with high O_3_ pollution, such as the Bohai Rim, North China Plain, the Yangtze River Delta urban agglomeration, and Central Inner Mongolia [11,12], and it has been concluded that the dominant factors driving O_3_ pollution also exhibit certain regional differences. For example, the spatial variability in O_3_ concentrations in the Yangtze River Delta urban agglomeration appeared to be mainly driven by socioeconomic factors such as the economic scale, urbanization, and emission sources [13]. Similarly, the temperature, wind speed, and the proportion of the GDP made up by secondary industry were all identified as important factors for O_3_ concentrations in North China [14]. However, most of the above studies on O_3_ concentrations focused on socioeconomic factors in densely populated areas, and relatively few focused on areas that can be regarded as having low levels of human activity. Therefore, more systematic studies on the spatiotemporal variability of O_3_ concentrations under the influence of natural factors such as climate and surface vegetation should be conducted.

In recent years, O_3_ pollution in Northeast China has become increasingly prominent, but the O_3_ concentration in the region varies greatly from north to south, with low values in the north and high values in the Southeastern Bohai Rim area adjacent to the high pollution center in North China [15]. This distribution demonstrates the importance of O_3_ sources and the urgent need to strengthen treatment efforts. By analyzing the evolution of spatial patterns and influencing factors of O_3_ concentrations in Northeast China in 2016, Chen et al. [16] found that temperature was the dominant factor determining annual average O_3_ concentrations. However, the spatial variability in temperature in Northeast China is large, so the annual differences among areas are significant. The factors driving O_3_ concentrations are found to be different in different regions, and the effect sizes of different factors will vary among seasons. This introduces high levels of complexity that greatly limit our understanding of the contributing mechanisms and our ability to predict O_3_ concentrations throughout Northeast China. Moreover, Northeast China has the largest natural forest area in China, and plants are known to produce high levels of bio-volatile organic compounds (BVOCs), which can act synergistically with precursors to enhance O_3_ production [17]. These large natural sources of BVOCs further complicate O_3_ pollution mechanisms in Northeast China. Therefore, based on the O_3_ concentration data; meteorological data (temperature, relative humidity, wind speed, sunshine hours, and precipitation); and green land area data in Northeast China from 2017 to 2020, the current study uses the spatial autocorrelation analysis, cold–hot spot analysis, geographical detector, and other spatial analysis methods to clarify the spatial–temporal variation rules of O_3_ concentrations in Northeast China. The main factors controlling O_3_ concentrations were comprehensively analyzed, while accounting for seasonal differences and spatial agglomeration patterns in O_3_ concentrations, to provide a scientific basis for the prevention and control of pollution in low O_3_ concentration areas.

## 2. Materials and Methods

### 2.1. Study Area

In this paper, Northeast China was selected as the study area, including Heilongjiang Province, Jilin Province, and Liaoning Province, as well as Hulunbuir, Chifeng, Tongliao, Hinggan League, and Xilin Gol League of the Inner Mongolia Autonomous Region, with a total area of about 1.52 million km^2^. The study area is bordered by North China to the south, Russia to the north, Japan across the sea to the east, and the Inner Mongolia Autonomous Region to the west. The terrain is mainly plains, hills, and mountains. The main geographical features include the northeast plains and the Greater Khingan Mountains, Lesser Khingan Mountains, and Changbai Mountains. The forest coverage in the study area was 47.2%, significantly higher than the national level (23.0%). This region is an important grain production area for China. The northern part of Northeast China is in a cold temperate zone, while the southern part is in a warm temperate zone. According to the China Meteorological Data Network, the annual temperature difference between the northern and southern portions was as high as 10.8 °C in 2020. In recent years, due to the increasing clean energy capacity in Northeast China, particulate pollutants such as PM_2.5_ and PM_10_ have been gradually decreasing, but ozone pollution has continued to increase [17].

### 2.2. Data Sources

**Figure 1:**
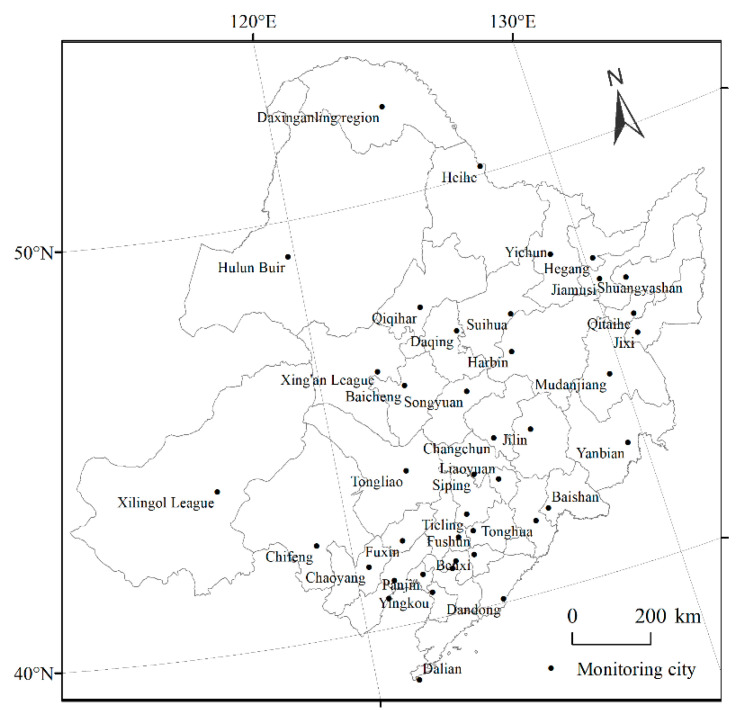
Sites of state-controlled monitoring stations in Northeast China.

The O_3_ concentration data, as well as the atmospheric pollutants (PM_2.5_, PM_10_, CO, SO_2_, and NO_2_) were obtained from 41 state-controlled monitoring stations in Northeast China (Figure 1). The original data were from the national urban air quality real-time publishing platform (https://www.mee.gov.cn/, accessed on 20 March 2021) and covered from 1 January 2017 to 28 February 2021. The O_3_ concentrations are monitored in real time by the National Environmental Monitoring Station, which uses sodium indigo disulfonate spectrophotometry and ultraviolet photometry. The O_3_ concentrations were analyzed on monthly and annual scales, and the meteorological determination method was used for the analysis of the seasonal variations. Spring, summer, autumn, and winter were defined as March–May, June–August, September–November, and December–February, respectively. The daily O_3_ evaluation index adopts the maximum 8-h sliding mean concentration of O_3_, which refers to the maximum of all the 8-h sliding mean concentrations from 8:00 to 24:00 in a natural day. The 90th percentile of the maximum 8-h moving average concentration of O_3_ was also measured. The maximum daily 8-h sliding average O_3_ concentration (O_3_-8 h) was used as the daily evaluation index, and the 90th percentile of the maximum daily 8-h sliding average O_3_ concentration (O_3_-8h-P90) was used as the annual evaluation index.

From 1 January 2017 to 28 February 2021, the cumulative daily ozone data provided by 41 monitoring stations was 69,577 days, and the validity and invalidity of the data were determined by the existence of the data. If data were missing, they were considered invalid; otherwise, the data were considered valid. The valid amount of O_3_ data was 59,397 d in total, with an effective rate of 99.16%. The evaluation standard was implemented according to the Technical Provisions of the Air Quality Index (AQI) (Trial) (HJ 633-2012), and the O_3_ concentrations were divided into 6 levels: excellent (0–100 μg·m^−3^), good (101–160 μg·m^−3^), lightly polluted (161–215 μg·m^−3^), moderately polluted (216–265 μg·m^−3^), heavily polluted (266–800 μg m^−3^), and severely polluted (>800 μg m^−3^). The green land area was set according to the China Urban Statistical Yearbook, and the meteorological data (temperature, relative humidity, wind speed, sunshine duration, and precipitation) were collected from China’s daily surface climate dataset (V3.0) released by the China Meteorological Data Network (http://data.cma.cn/site, accessed on 20 March 2021). The data have been rigorously screened in previous applications and shown to be robust.

### 2.3. Methods

#### 2.3.1. Spatial Autocorrelation Analysis

According to the first law of geography, spatial distributions are geographically interdependent within regions, and the closer the distance, the stronger the correlation—that is, variables exhibit spatial autocorrelation [18,19,20]. Spatial autocorrelation can be used to reveal spatial dependencies and the heterogeneity of observed variables in a study area. In this paper, Moran’s *I* was used as the autocorrelation index for O_3_ concentrations in monitored cities in Northeast China. *I* is a value within [−1, 1], where *I* > 0 indicates that the O_3_ concentration has a positive spatial correlation, *I* < 0 indicates a negative spatial correlation, and *I* = 0 indicates a random distribution.

#### 2.3.2. Cold–Hot Spot Analysis

Global spatial autocorrelation (Moran’s *I*) can reflect the overall aggregation or dispersion in a regional space but cannot reflect locally specific conditions. In the current research, the Getis–Ord (G_i_*) cold–hot spot analysis was used to assess the O_3_-8 h-P90 aggregation characteristics in Northeast China in 2017–2020. If G_i_* exceeds the 90% confidence level, it indicates that the area and surrounding areas are hot zones that have high O_3_ concentrations; otherwise, the areas are identified as cold spots with low O_3_ concentrations. Areas that fail the significance test are said to exhibit no spatial aggregation characteristics and show discrete distributions. The calculation result of G_i_* is the *Z* value, and the higher the *Z* value, the tighter the clustering of a hot spot area is; conversely, the lower the *Z* value, the tighter the clustering of a cold spot area [21].

#### 2.3.3. Geographical Detector

A geographical detector is a statistical tool for quantifying the factors influencing the spatial differentiation of geographical objects. This model makes up for the shortcomings of traditional statistical methods by detecting the influences of independent variables on dependent variables using the relationships between the intra-level variance and global variance of each variable, i.e., the spatial differences in the degree of influence of independent variable *X* on dependent variable *Y* [12]. The current research considered both seasonal differences and spatial clustering patterns in O_3_ concentrations. The factor detection tool in the geographical detector was used to identify the driving forces and magnitude of spatial differentiation among O_3_ concentrations, meteorological factors, and green land area, and the influence directions of the factors were analyzed by a correlation analysis.

## 3. Results

### 3.1. Temporal Variation in O_3_ Concentrations

#### 3.1.1. Annual Variation

**Figure 2:**
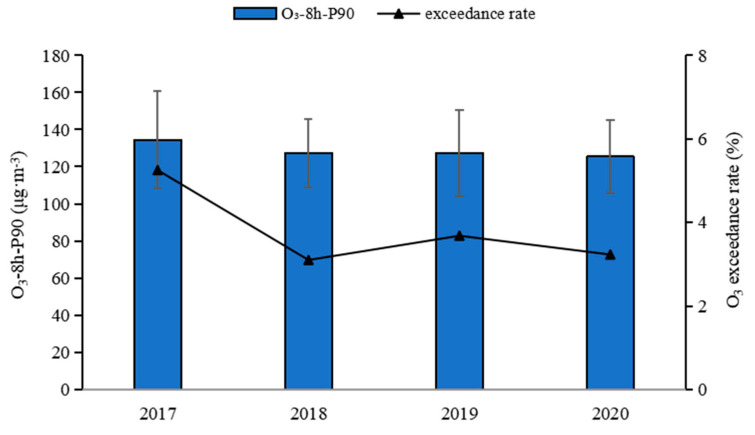
Variations in O_3_-8 h-P90 and the over-standard rate from 2017 to 2020 in Northeast China.

The mean annual concentrations of O_3_-8 h-P90 in Northeast China from 2017 to 2020 were 134 μg·m^−3^, 127 μg·m^−3^, 127 μg·m^−3^, and 125 μg·m^−3^, respectively (Figure 2), which were lower than the national secondary standard (160 μg·m^−3^). Compared with 337 cities in China, this region was 12.5% lower than average, making it a low O_3_ concentration area. On the whole, the O_3_ concentration decreased by 6.7% during 2017–2020. However, the variance analysis showed that there was no significant change in the O_3_ concentration during the 4 years (*p* = 0.265), so the average O_3_ concentration could represent the overall pollution level in the recent years. Our result was slightly lower than the O_3_ concentration reported by [22] from 2015 to 2018 (131–138 μg·m^−3^), which was mainly due to the scope of the study area. Five league cities with low O_3_ concentrations (annual average of 82 μg·m^−3^), including Chifeng, Tongliao, Hulunbuir, Hinggan League, and Xilin Gol League, were included in the present study. In terms of O_3_-8 h in Northeast China (Figure 2), the annual over-standard rates (O_3_-8 h concentrations greater than secondary standards) were 5.25%, 3.1%, 3.68%, and 3.23% from 2017 to 2020, respectively, with the O_3_ pollution conditions being mainly categorized as excellent and good. In the past four years, the O_3_ concentration has exhibited the same trend as the over-standard rate, roughly decreasing each year, suggesting that O_3_ pollution control has had a notable impact.

#### 3.1.2. Seasonal Variation

**Figure 3:**
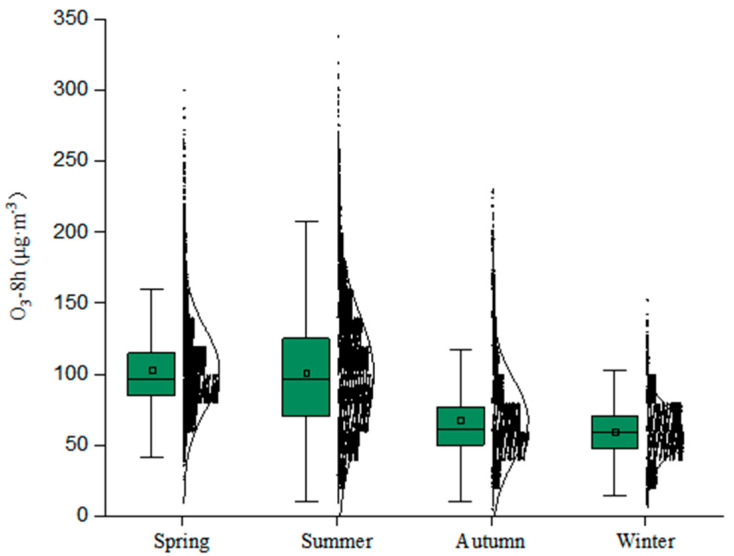
Seasonal variation in of O_3_-8 h from 2017 to 2020 in Northeast China. (The middle horizontal line of the boxplot represents the median O_3_ concentration; the top and bottom of the box are the top quartile and the bottom quartile. The scatter plot to the right of the boxplot represents the distribution of O_3_ concentration, and the curve represents the trend of O_3_ concentration).

Figure 3 shows the distribution of O_3_-8 h concentrations in the 41 monitored cities in Northeast China from 2017 to 2020 in different seasons. The concentrations ranged within 26–300 μg·m^−3^ in the spring, 10–338 μg·m^−3^ in the summer, 5–230 μg·m^−3^ in the autumn, and 8–152 μg·m^−3^ in the winter, with the largest variability occurring in the summer and the smallest in the winter. The highest seasonal average concentration of O_3_-8 h was 103 μg·m^−3^ in the spring, followed by 101 μg·m^−3^ in the summer, 68 μg·m^−3^ in the autumn, and 59 μg·m^−3^ in the winter. The highest value (338 μg·m^−3^) occurred in the summer, exceeding the national O_3_ quality standard IV (266 μg·m^−3^) and reaching the severe pollution level. Except the winter, pollution in the other seasons was categorized as light and above. The over-standard rate was 8.51% in the summer, 5.24% in the spring, and 1.24% in the autumn, and there was no over-standard phenomenon in the winter. The proportion of clean days exceeded 98% in both the autumn and winter, with moderate and high levels of pollution occurring only in the spring and summer. In general, the seasonal O_3_-8 h concentrations in Northeast China from 2017 to 2020 were characterized by high concentrations in the spring and summer and low concentrations in the autumn and winter. This was mainly due to the high summer temperatures, strong solar radiation, and strong photochemical reactions between ozone and its precursors, which have a catalytic effect on ozone production [23].

#### 3.1.3. Monthly Variation

**Figure 4:**
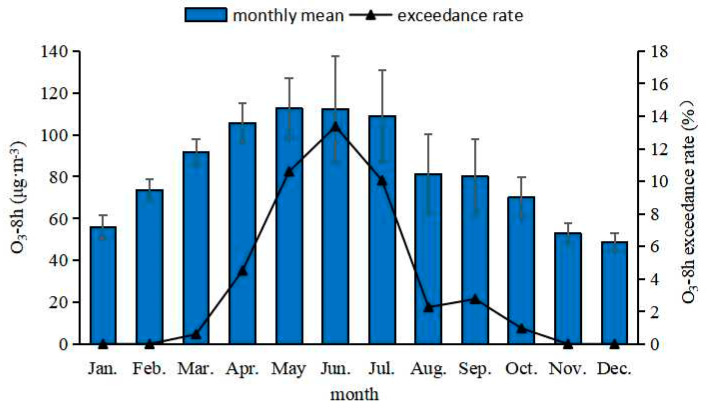
Monthly concentrations and over-standard rates of O_3_-8 h from 2017 to 2020 in Northeast China.

From 2017 to 2020, the month-to-month trends in O_3_-8 h concentrations in Northeast China showed obvious inverted “V” patterns of first increasing and then decreasing during each year (Figure 4). The O_3_ concentration increased gradually from January to May, with a monthly increase of 0.98 μg·m^−3^ followed by a slow monthly decrease of 0.955 μg·m^−3^ from June to December. The highest O_3_ concentration was 2.3 times the lowest value. The monthly average over-standard rates in the past 4 years demonstrated that the changes of O_3_ concentrations and over-standard rates in Northeast China have been generally consistent. The O_3_ over-standard rates from May to July were the highest at 10.6%, 13.4%, and 10.1%, respectively. The over-standard rates from March to April and August to October were all lower than 5%. It is worth noting that O_3_ pollution did not exceed the standard in January–February and November–December, indicating that O_3_ generation was slow in late autumn and the winter in Northeast China. This may have been because the temperatures in Northeast China during these periods are extremely low and the daily sunshine is limited, thus inhibiting the generation of O_3_. O_3_ is generated by a photochemical reaction with VOC_S_, NO_X_, and other precursors under the action of solar radiation, and its reaction speed is positively correlated with temperature. In late autumn, the temperature drops to below 0 °C, and with the temperature decreasing, the photochemical reaction speed slows down accordingly, so the generation of ozone is slow. In addition, precipitation in Northeast China is relatively high in the winter, which can enhance the settling and clearing of atmospheric O_3_ [16].

### 3.2. Spatial Variability in O_3_ Concentrations

#### 3.2.1. Annual Variation

**Figure 5:**
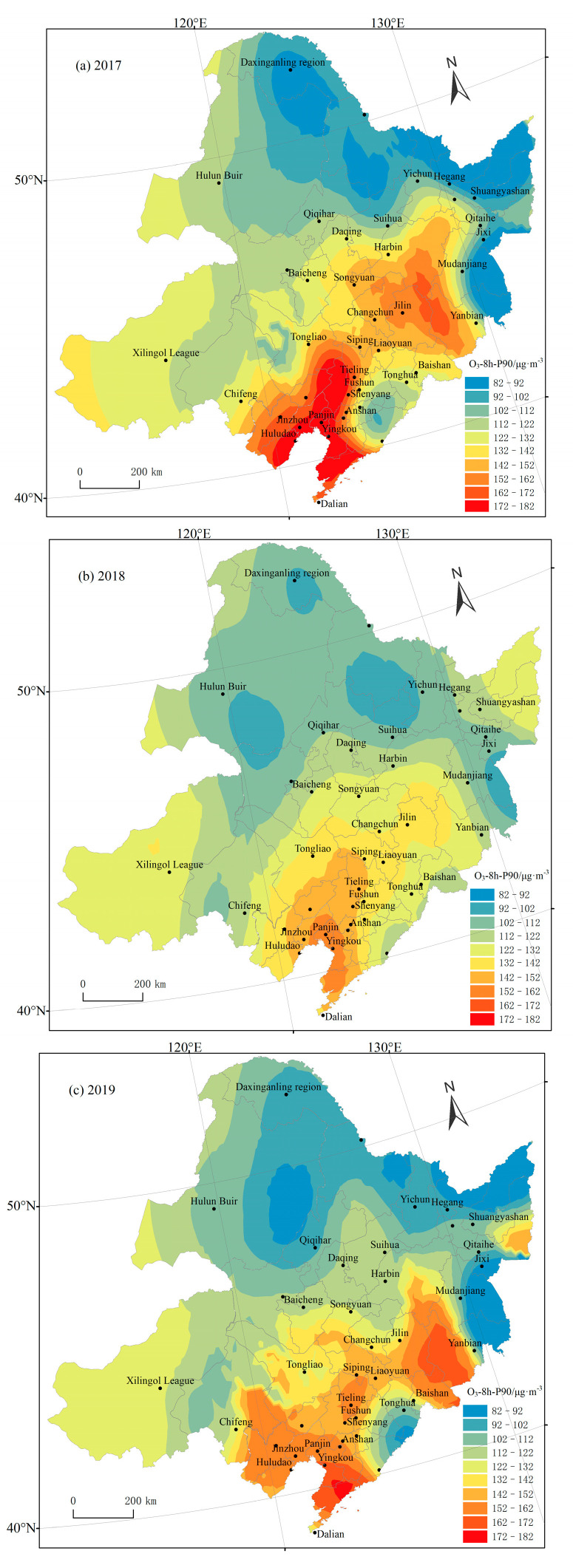

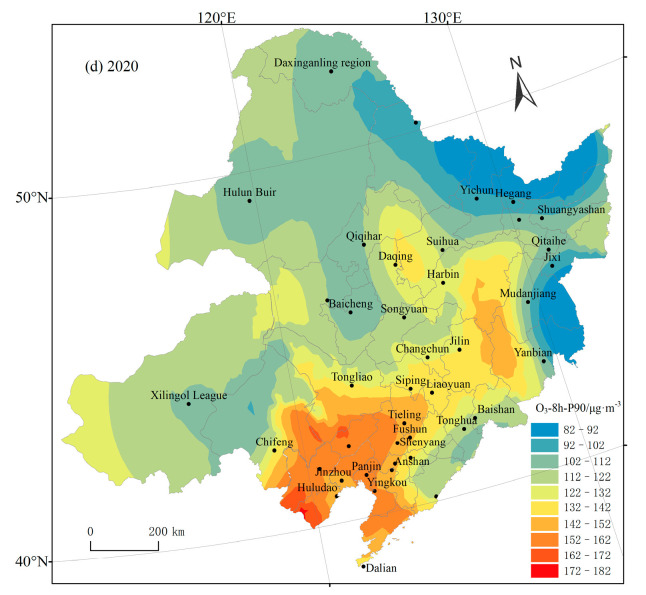
Annual spatial distributions of O_3_ concentrations from 2017 to 2020 in Northeast China.

From 2017 to 2020, the spatial distributions of the O_3_-8 h-P90 concentrations in Northeast China were relatively consistent (Figure 5), with the overall characteristics of high concentrations in the southeast, low concentrations in the northeast, and moderate concentrations in the west. The O_3_ concentrations were highest in Liaoning Province, followed by Jilin and Heilongjiang Provinces, and lowest in the five cities in the Inner Mongolia Autonomous Region. The northeast region of China is located in the typical monsoon climate area, and the northwest wind prevails in the winter. Compared with the southern region, the demand of coal burning for heating is greater in the northern region. The nitrogen oxides generated in the process of coal burning are conducive to the generation and accumulation of ozone, and ozone and other pollutants are transported to the southern region under the action of the prevailing wind. Therefore, the south of the northeast area is the downwind area that transmits the most serious ozone pollution. In 2017, the highest concentrations of O_3_ were located in the Southeastern Bohai Rim area and connected with the smaller pollution area in Jilin, Jilin Province, forming the high incidence area of O_3_ pollution. The O_3_ concentrations of 11 cities, including Shenyang, Jinzhou, Huludao, Panjin, Yingkou, and Jilin, were higher than the national second-class standard. In 2018, O_3_ pollution started to be alleviated, and the low-value area gradually expanded. Compared with the O_3_ concentrations in 2017, the average value of O_3_ decreased by 10.3% in Liaoning Province, 7% in Jilin Province, and 5% in the five leagues of the Inner Mongolia Autonomous Region but increased by 2% in Heilongjiang Province. In 2019, the high O_3_ pollution area extended from Liaoning Province to Liaoyuan, Siping, and Yanbian in Jilin Province, and the average O_3_ in Liaoning Province and the five cities in the Inner Mongolia Autonomous Region increased by varying degrees. In 2020, the O_3_ concentration in Jilin Province, Liaoning Province, and the five cities of the Inner Mongolia Autonomous Region showed a downward trend, but the O_3_ concentration in Heilongjiang Province increased from 10_3_ μg·m^−3^ in 2019 to 107 μg·m^−3^ in 2020. In general, the high O_3_ pollution areas in Northeast China were contiguously distributed, and cohesive regional prevention and control efforts need to be strengthened. Furthermore, the O_3_ concentrations in low-value pollution areas increased in 2020, indicating that the relevant authorities need to increase their efforts to combat O_3_ pollution in the low-value areas as well.

#### 3.2.2. Seasonal Variation

**Figure 6:**
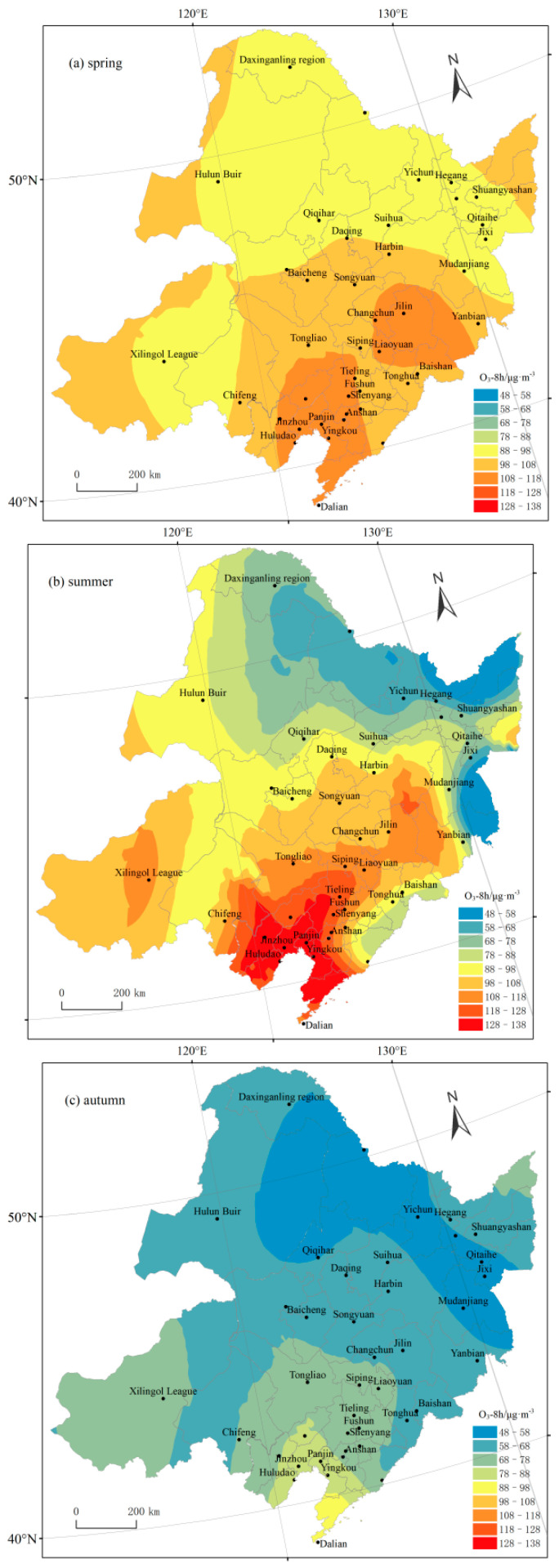

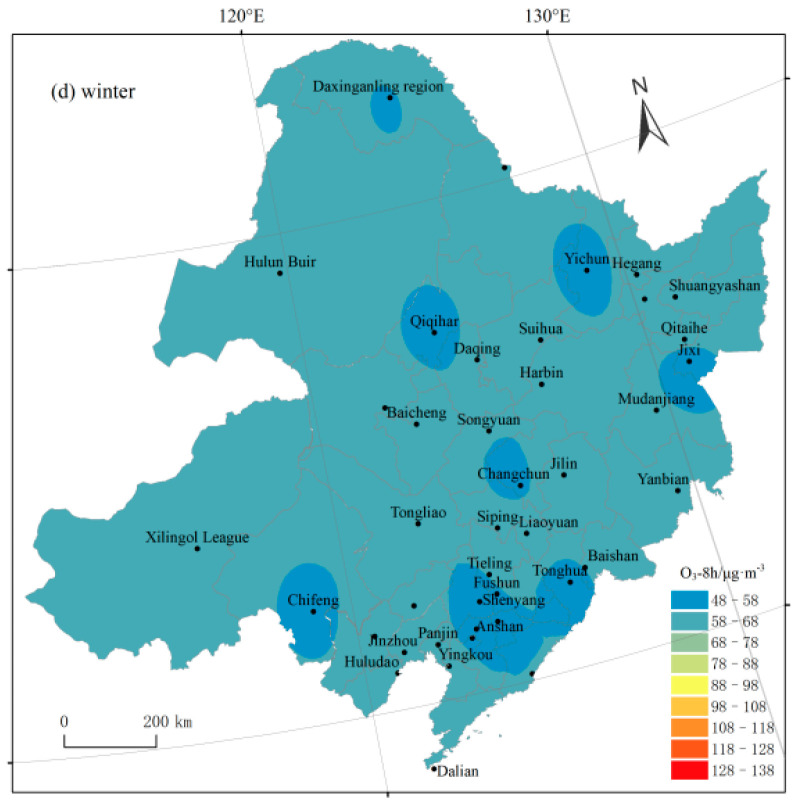
Seasonal spatial distributions of O_3_ concentrations from 2017 to 2020 in Northeast China.

The spatial distributions of O_3_-8 h concentrations in Northeast China changed significantly among the seasons (Figure 6), and the variation coefficients in the spring, summer, autumn, and winter were 9.03%, 21.5%, 15.34%, and 6.75%, respectively. In the summer, O_3_ pollution was the most serious, and the spatial variation was most pronounced, with the highest value being 2.2 times the lowest value. The main reason for the most serious O_3_ pollution in the summer is the long sunshine time in the summer, the strong solar radiation, and the high temperature accelerates the photochemical reaction of ozone, so that the ozone concentration rises. The O_3_ concentrations gradually increased from north to south, with the highest concentration in Liaoning Province (123 μg·m^−3^), followed by Jilin Province (101 μg·m^−3^) and the five cities of the Inner Mongolia Autonomous Region (102 μg·m^−3^), and the lowest concentration in Heilongjiang Province (77 μg·m^−3^). In the autumn, the O_3_ concentrations decreased by 35% in Heilongjiang Province, 53% in Jilin Province, 58% in Liaoning Province, and 52% in the five cities of the Inner Mongolia Autonomous Region. In the autumn, the spatial differentiation of the O_3_ concentrations weakened in the low-value areas in the north but still exhibited high spatial variability in the high-value areas in the south. The O_3_ concentrations in the spring were generally higher than in the autumn, but the spatial distributions were generally similar. The average O_3_ concentrations in the winter were the lowest, when the spatial variation was also the smallest, in the range of 51–69 μg·m^−3^. The main reason for the seasonal spatial variability in the O_3_ concentrations was the large temperature difference between the north and south in the summer and the low average temperature in the winter in Northeast China. This agrees with previous reports of a positive correlation between temperature and O_3_ concentrations.

### 3.3. Spatial Aggregation Characteristics of O_3_ Concentration

A global spatial autocorrelation analysis of the O_3_-8 h-P90 concentrations in the 41 monitoring stations in Northeast China from 2017 to 2020 was conducted. The results showed that, from 2017 to 2020, the annual Moran’s I index values were all positive (10.8, 10.41, 12.55, and 12.53, respectively), and the *Z*(*I*) values were all greater than 2.58. This indicated that the O_3_-8 h-P90 concentration in Northeast China was significantly positively autocorrelated and exhibited obvious spatial agglomeration characteristics.

**Figure 7:**
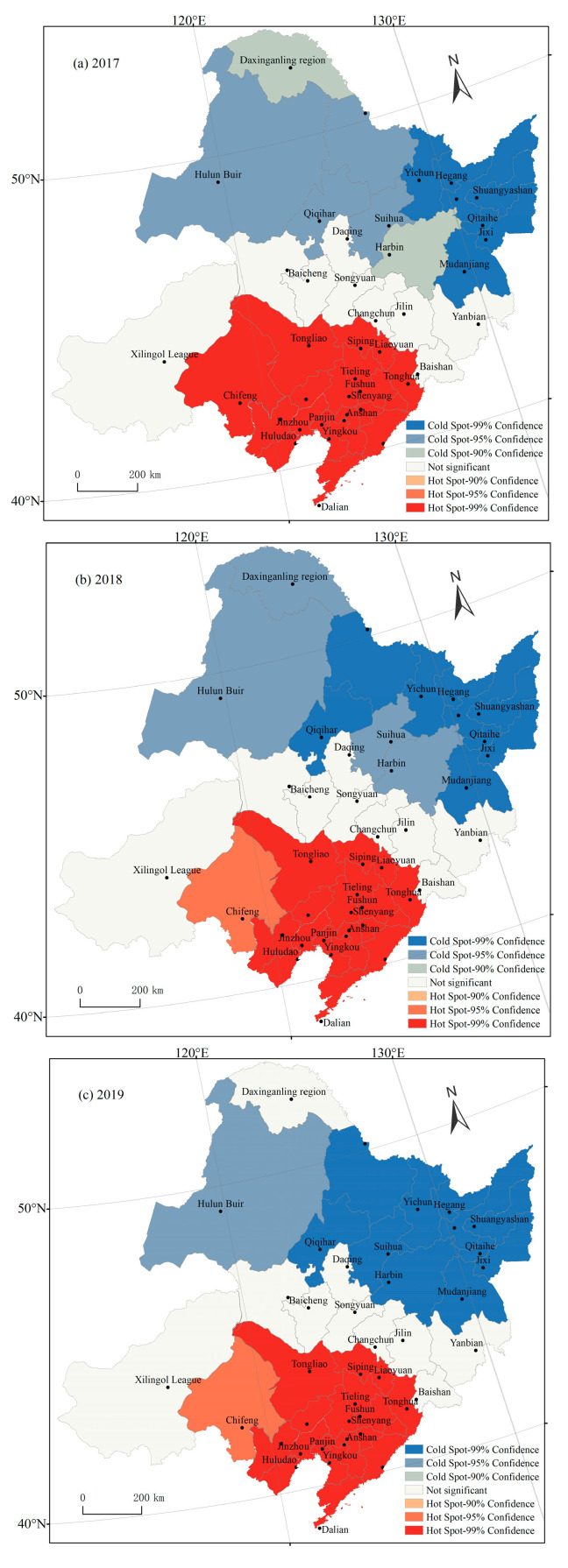

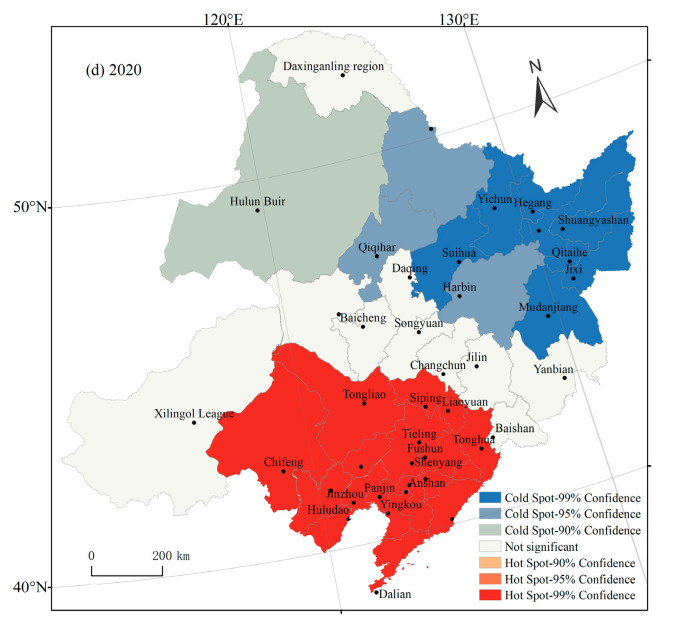
Interannual evolution of the spatial concentrations of O_3_ from 2017 to 2020 in Northeast China.

The cold–hot spot analysis method was used to further reveal the spatial agglomeration characteristics of O_3_-8 h-P90 concentrations in Northeast China during the past four years (Figure 7). The results showed that the O_3_-8 h-P90 concentrations in Northeast China exhibit the spatial agglomeration characteristics of being hot in the south and cold in the north. In 2017, the hot spots were mainly distributed in Liaoning Province; Siping, Liaoyuan, and Tonghua in Jilin Province; and Chifeng and Tongliao in the Inner Mongolia Autonomous Region, reflecting the homogenization of high–high agglomerations due to the high O_3_ concentrations in these areas. The cold spots were located in Hulunbuir of the Inner Mongolia Autonomous Region and 12 cities in Heilongjiang Province, except Harbin, where the O_3_ concentrations were low, showing the homogenization of low–low agglomerations. However, the feature-free spots were mainly distributed in the transition regions between cold and hot spots, where the spatial aggregations of the O_3_ concentrations were relatively scattered with weak correlations. The cold and hot spots in 2017 and 2018 were exactly the same. However, in 2019 and 2020, only the hot spots were the same as the previous two years, while the cold spots were smaller. In conclusion, the hot spots of the O_3_ concentrations in Northeast China did not change significantly from 2017 to 2020, but there was a trend in which the cold spots transitioned to feature-free spots.

## 4. Discussion

### 4.1. Relationships among O_3_, Meteorological Factors, and Atmospheric Pollutants

**Figure 8:**
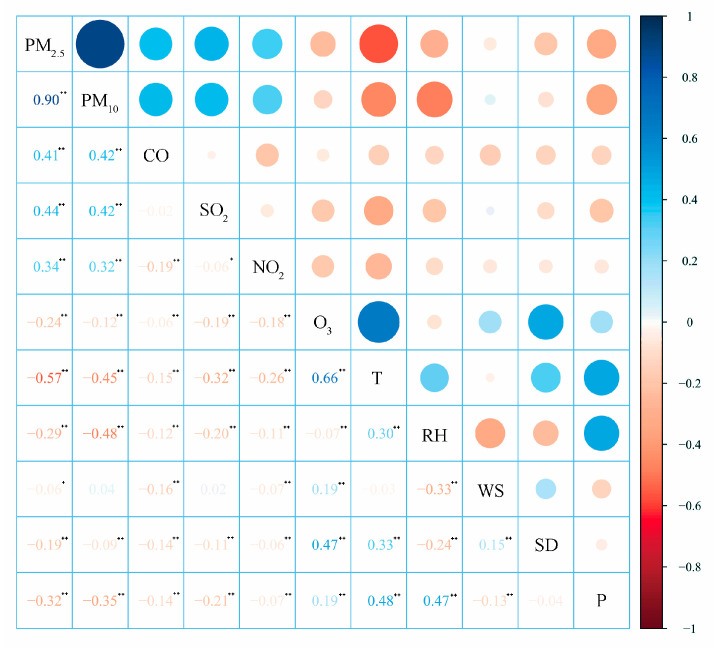
Correlation coefficients among meteorological factors and atmospheric pollutants. (T: temperature, RH: relative humidity, WS: wind speed, SD: sunshine duration, and P: precipitation. ** Indicates that the correlation is significant at the level of 0.01, and * indicates that the correlation is significant at the level of 0.05.)

The tropospheric O_3_ concentrations were effected by natural meteorological factors and the release of precursors from human activities [23]. The relationships of the O_3_ concentrations and all the meteorological factors (temperature, relative humidity, wind speed, sunshine duration, and precipitation), as well ae atmospheric pollutants (PM_2.5_, PM_10_, CO, SO_2_, and NO_2_) during 2017–2020, were investigated to evaluate the effects of each meteorological factor and atmospheric pollutant on O_3_ pollution in Northeast China (Figure 8), especially the effects of temperature and O_3_ precursors.

The results show that O_3_ has a significant negative correlation with other pollutants such as PM_2.5_, PM_10_, CO, SO_2_, and NO_2_. The correlation order is PM_2.5_ > SO_2_ > NO_2_ > PM_10_ > CO. The decrease of other pollutants leads to the increase of O_3_ concentration. O_3_ is positively correlated with temperature, wind speed, sunshine duration, and precipitation among the meteorological factors and negatively correlated with the relative humidity, all of which pass the reliability test at the level of 0.01. Temperature is the dominant factor affecting O_3_ concentration, and the correlation coefficient is 0.66. The higher the temperature, the stronger the photochemical reaction between O_3_ and its precursors, and the faster the O_3_ generation, followed by the sunshine duration. The influence of sunshine duration on the O_3_ concentration is mainly reflected in solar radiation, and studies have shown that strong solar radiation is the key factor leading to O_3_ rise. The sunshine duration is closely related to solar radiation, so there is a positive correlation between sunshine duration and O_3_. The weak correlation between wind speed and precipitation and O_3_ may be due to the fact that the average annual wind speed in Northeast China is 2.6 m/s, and the average annual precipitation is only 11 mm. Low wind speed and precipitation have no significant influence on the change in O_3_ concentration. The relative humidity has a negative influence on O_3_, and the correlation is not strong. High humidity scours and clears the O_3_, resulting in a low O_3_ concentration.

### 4.2. Seasonal Effects of Meteorological Factors and Precursor on O_3_

There were significant spatial differences in the O_3_ concentrations in Northeast China, which could be generalized as high pollution areas in the south and low-value areas in the north. From the south to the north, the spatial agglomeration characteristics transitioned from hot spots to feature-free areas and then to cold spots. This pattern provided a good spatial gradient for further revealing the dominant factors influencing O_3_ pollution at different locations. Compared with Huang-Huai and middle-lower reaches of the Yangtze River, which are densely populated and economically developed, temperature plays a larger role in the O_3_ distribution in Northeast China. The temperature gradient in Northeast China is large, and the seasonal differences are significantly larger than those in other regions. The average temperature in the summer is 20.9 °C higher than that in the winter. The temporal changes in the O_3_ concentrations reflect the temperature trends, i.e., higher in the spring and lower in the autumn and winter, which provides a valuable research opportunity for clarifying the mechanism by which temperature controls the generation of O_3_. Therefore, this study used a geographical detector to detect spatial differentiation characteristics and rules. Meteorological factors such as temperature, relative humidity, wind speed, sunshine duration, precipitation, and vegetation coverage were examined. The factor detector was selected to determine which factors were driving the variability in the O_3_-8 h concentrations in Northeast China from 2017 to 2020 at both the seasonal and regional levels. Meanwhile, in combination with the correlation analyses between each explanatory variable and O_3_ concentration, the positive and negative effects of the influencing factors were clarified.

In the present research, the O_3_ concentration data; meteorological data (temperature, relative humidity, wind speed, sunshine hours, and precipitation); and green land area data were investigated to deeply clarify the impacts of meteorological and vegetation factors on the spatial–temporal variability of O_3_ concentrations in Northeast China from 2017 to 2020. The seasonal analysis of the influencing factors showed (Table 1) that the temperature, relative humidity, and precipitation had significant influences (*p* < 0.05), but there were obvious seasonal differences in their degrees of effect. However, sunshine duration and wind speed failed the significance test. This indicated that, when the O_3_ concentration in the region was relatively low, the sunshine duration had no obvious effect on O_3_ generation and neither did wind diffusion. Temperature had the largest effect on O_3_, and its explanatory power *q* values were 0.345, 0.205, and 0.294 in the spring, summer, and autumn, respectively (*p* < 0.05). The average temperatures in the spring and autumn in Northeast China ranged within 7.4–8.7 °C, and precipitation was relatively low. Within this temperature range, temperature was the main influencing factor controlling O_3_ generation. However, the explanatory power of temperature decreased in the summer. This was due to strong air convection and high precipitation in the summer, which play important roles in scouring and clearing accumulated O_3_ [22]. Therefore, the influence of temperature in the summer is weakened by relative humidity and precipitation. It is worth noting that the O_3_ concentration in the winter was not affected by meteorological factors, with all factors exhibiting weak explanatory powers. This was because the winter in Northeast China is cold and long, the average annual temperature is about −11 °C, and the solar radiation is weak, which means the photochemical reactions of O_3_ precursors and O_3_ generation are slowed. The O_3_ concentrations in the winter were mostly classified as excellent. Based on the above observations, when the monthly mean temperature in Northeast China ranges between −2.2 and 16.5 °C, and the monthly precipitation in the quarter ranges between 5 and 1098 mm, the temperature will play a positive role in promoting O_3_ generation. When the monthly mean temperature is higher than 22.4 °C, and the monthly precipitation in the quarter ranges between 50 and 2559 mm, the O_3_ concentration will be positively correlated with the temperature but negatively correlated with precipitation. The wet deposition exhibited some influence on removing O_3_, while the wind speed and sunshine duration had little influence on the spatial distribution of the O_3_ concentrations.

The NO_2_ seasonal geographic detection results showed that all seasons passed the significance test except the summer, and the *q* values of influence in the spring, autumn, and winter were −0.104, −0.153, and −0.217, respectively, indicating that the increase in ozone concentration was related to the decrease in nitrogen dioxide. When the concentration of O_3_ was between 58 μg·m^−3^ and 68 μg·m^−3^, the influence of NO_2_ on O_3_ was the most prominent, accounting for 42%. When the concentration of O_3_ was higher than 68 μg·m^−3^ and lower than 100 μg·m^−3^, the correlation was weak, and the higher the concentration was, the weaker the correlation. In general, the influence of NO_2_ on the distribution pattern of the O_3_ concentration in Northeast China was negative, and the mean value of q was −0.108. This result may be caused by the overall low concentration of O_3_ in Northeast China, compared with other regions of China; the intensity of the human activities in Northeast China is weak, so the precursors released by human activities have little influence on O_3_.

### 4.3. Effects of Meteorological Factors, Green Land Area, and Precursor on Different O_3_ Pollution Levels

All factors influencing cold and hot spots according to the geographical detector are provided in Table 2. The influences of temperature and green land area on the O_3_ concentration were positive (*p* < 0.05), while the influence of relative humidity was negative (*p* ≤ 0.05). Wind speed, sunshine duration, and precipitation failed the significance tests, indicating they had weak or no explanatory power over the spatial distributions of O_3_. The influence of temperature was consistent with the seasonal geographic survey findings. When the average annual temperature was 4.9–11.9 °C, temperature was the main factor controlling O_3_ distributions, and the higher the average annual temperature, the stronger the influence of the temperature. When the average annual temperature was lower than 3.3 °C, the effect of temperature was not significant. This may have been because the high temperature and strong solar radiation accelerated the photochemical reactions of VOCs, NOx, and O_3_ and other precursors, leading to increased O_3_ concentrations. The relative humidity in Northeast China increased from south to north. In the feature-free spots and cold spots (relative humidity 58–63%), the relative humidity had a significant negative correlation with the O_3_ concentration. In hot spots, the relative humidity had no significant effect, and the O_3_ concentration was mainly affected by the temperature. In cold spots, the relative humidity was the main factor influencing the O_3_ concentrations. Similarly, the green land area had a positive relationship with cold spots and feature-free spots. Vegetation can release a large amount of natural VOCs, which synergistically interact with NOx to promote the generation of O_3_. This was especially apparent in Changchun, Daqing, and other places with high vegetation coverage, where the green land and temperature jointly dominated the spatial distribution of O_3_. Li et al. [24] also found that vegetation coverage was closely related to the O_3_ concentration, and the O_3_ concentration changes corresponded well with the properties of the underlying surface. The regional NO_2_ geographical detection results showed that the hot spot and cold spot did not pass the significance test, indicating that there was no significant correlation between NO_2_ and O_3_ in the high and low O_3_ concentration areas, and there was a negative correlation between O_3_ and NO_2_ at the level of 0.05 in the featured-point areas. The increase of NO_2_ will reduce O_3_. In general, the concentration of O_3_ in Northeast China is weakly affected by precursors such as NO_2_, and its variation is mainly affected by meteorological factors such as temperature, relative humidity, and precipitation.

Therefore, in hot spots with high O_3_ concentrations, temperature was the predominant controlling factor, but other factors made significant contributions. With the decline in the O_3_ concentration, the effect of temperature gradually weakened, and the influence of the relative humidity and green coverage area showed increasing trends. In cold spots with low O_3_ concentrations, the spatial distributions of O_3_ were affected by both the relative humidity and green land coverage area.

The spatial distribution of ozone in Northeast China is the result of both natural and human factors [25,26,27]. Natural factors mainly refer to meteorological factors. High temperatures can promote the generation of ozone. Global climate warming causes frequent abnormal atmospheric warming, and the higher the temperature, the faster the chemical reaction of ozone [28]. We will vigorously develop renewable energy. The reduction in greenhouse gas emissions has lowered the temperatures and, thus, ozone levels across the Earth’s surface [29]. Human factors mainly refer to the increase of VOC emissions under the interference of human activities. The sources of VOC_S_ are very complex, most of which come from the emissions of traffic sources, living sources, and industrial sources. Therefore, the country should improve the VOC monitoring system and increase the control of VOC_S_. We can learn from the advanced experience of the United States to implement more stringent emission policies and environmental tax incentives for industrial enterprises, such as the installation of energy-saving equipment and enterprises enjoying tax credits, so as to achieve the reduction of the ozone precursor VOC content and then reduce the ozone concentration in a certain space. In conclusion, the national mitigation strategy for the ozone concentration should focus on the source control and the end control. In the end control, a perfect ozone pollution monitoring and control system should be established. In this respect, the PAMS photochemical monitoring network in the United States is of great reference value.

If the ozone increases in the future, the number of people exposed to ozone and suffer health risks will increase, and the mortality rate of our population will increase due to acute lower respiratory tract infections, lung cancer, ischemic heart disease, and stroke, which will directly cause very serious economic losses [30,31]. Ozone pollution will also have an adverse effect on the growth of crops. China is a large grain production country. The increase of the ozone content will lead to the decrease of grain production of wheat and rice, and the food supply will be threatened [32,33]. In the future, air pollution control policies with higher targets should be formulated to further curb the rising trend of ozone, so as to fully guarantee public health and food security.

## 5. Conclusions

Generally, the O_3_ concentration of Northeast China were lower than the national secondary standards from 2017 to 2020, which belonged to a low O_3_ concentration region, suggesting that O_3_ pollution control has had a notable impact. The spatial distributions of the O_3_-8 h-P90 concentrations were relatively consistent, with the overall characteristics of high concentrations in the southeast, low concentrations in the northeast, and moderate concentrations in the west. O_3_ pollution was the most serious and the spatial variation was most pronounced in the summer, with the highest value being 2.2 times the lowest value. The main reason for that was the large temperature difference between the north and south in the summer and the low average temperature in the winter in Northeast China. The O_3_ concentrations exhibited the spatial agglomeration characteristics of being hot in the south and cold in the north. The feature-free spots were mainly distributed in the transition regions between cold and hot spots, where the spatial aggregations of O_3_ concentrations were relatively scattered with weak correlations.

The seasonal analysis of influencing factors indicated that the temperature, relative humidity, and precipitation had significant influences, while the sunshine duration and wind speed failed the significance test. The average temperatures in the spring and autumn in Northeast China ranged within 7.4–8.7 °C, and the precipitation was relatively low. Within this temperature range, the temperature was the main influencing factor controlling O_3_ generation. When the average summer temperature is between 6.3 and 33.1 °C, the ozone concentration increases in the spatial range. When the average winter temperature is between −37.6 °C and 11.1 °C, the spatial distribution of the ozone concentration decreases. A regional analysis of the influence factors showed that the influence of temperature and green space area on the O_3_ concentration was positive, and the influence of relative humidity on the O_3_ concentration was negative. The influences of temperature and green land area on the O_3_ concentration were positive, while the influence of relative humidity was negative. Wind speed, sunshine duration, and precipitation failed the significance tests, indicating they had weak or no explanatory power for the spatial distributions of O_3_.

## Figures and Tables

**Table 1:** Seasonal geographical detection of the driving factors.

	Temperature	Relative Humidity	Wind Speed	Sunshine Duration	Precipitation	NO_2_ Concentrations
Spring	0.345 **	−0.090	0.058	0.004	−0.077	−0.104 *
Summer	0.205 *	−0.252 *	0.027	0.091	−0.136 *	0.042
Autumn	0.294 *	0.073	0.036	0.017	−0.042	−0.153 *
Winter	0.067	−0.082	0.043	0.011	−0.015	−0.217 **

Note: ** and * indicate passing the significance test with reliabilities of 99% and 95%, respectively.

**Table 2:** Geographical detection analysis of all influencing factors in the cold and hot spots.

	Temperature	Relative Humidity	Wind Speed	Sunshine Duration	Precipitation	Green Land Area	NO_2_ Concentrations
Cold spots	0.063	−0.176 *	0.104	0.002	−0.043	0.137 *	−0.041
Feature-free spots	0.258 *	−0.126 *	0.006	0.038	0.045	0.282 *	−0.108 *
Hot spots	0.362 **	0.021	0.02	0.032	−0.028	0.06	−0.068

Note: ** and * indicate passing the significance test with reliabilities of 99% and 95%, respectively.

## Data Availability

The data used to support the findings of this study are available from the corresponding author upon request.
